# Facial Emotion Recognition and Emotional Memory From the Ovarian-Hormone Perspective: A Systematic Review

**DOI:** 10.3389/fpsyg.2021.641250

**Published:** 2021-05-20

**Authors:** Dali Gamsakhurdashvili, Martin I. Antov, Ursula Stockhorst

**Affiliations:** Experimental Psychology II and Biological Psychology, Institute of Psychology, University of Osnabrück, Osnabrück, Germany

**Keywords:** emotional memory, emotion recognition, menstrual cycle, estrogen, progesterone, oral contraceptives, ovarian hormones, sex hormones

## Abstract

**Background:**

We review original papers on ovarian-hormone status in two areas of emotional processing: facial emotion recognition and emotional memory. Ovarian-hormone status is operationalized by the levels of the steroid sex hormones 17β-estradiol (E2) and progesterone (P4), fluctuating over the natural menstrual cycle and suppressed under oral contraceptive (OCs) use. We extend previous reviews addressing single areas of emotional processing. Moreover, we systematically examine the role of stimulus features such as emotion type or stimulus valence and aim at elucidating factors that reconcile the inconsistent results.

**Methods:**

We followed the *Preferred Reporting Items for Systematic Reviews and Meta-Analyses* (PRISMA) guidelines and included papers published until September 2020 indexed in PubMed and Web of Science databases. Search terms were MeSH terms (emotional OR emotion) AND (*X*) AND (estrogen OR progesterone OR menstrual cycle OR oral contraceptives) with (*X*) representing our separately searched areas, resulting in (processing OR recognition OR empathy), and (memory OR recall). To be included, articles had to (1) be written and published in English, (2) examine healthy, non-pregnant adult women in their reproductive age, and (3) measure or at least estimate levels of E2 and P4. In *PubMed*, the search was (4) limited to humans and (5) to the search term present in the title or abstract.

**Results:**

Features of the provided stimulus material (emotion type and/or valence) constitute a relevant influence that interacts with E2- and P4-related ovarian-hormone status. For instance, recognition of basic emotions appears to be more related to P4- than E2-levels. Quite consistent, OC intake (vs. natural menstrual cycling) was accompanied by impaired recognition accuracy of basic and also complex emotions, although not in a recent large-sample study assessing complex emotions. Memory recall of negative content was mainly enhanced by P4, especially after having been stressed.

**Discussion and Conclusion:**

We document the methodological diversity in the field, presumably contributing to the heterogeneity of results. More studies explicitly contrasting the early follicular phase, mid-cycle phase, mid-luteal, and OC intake while standardizing tasks are needed. Research would take advantage of using within-subject designs and accounting for the recognition of complex emotions.

## Introduction

A growing number of original and review papers on the role of naturally fluctuating ovarian hormones and oral contraceptive (OCs) use for emotional processing indicates an increased interest in this field (for reviews see: Andreano et al., 2018; Osório et al., 2018; Lewis et al., 2019). Due to significant level variation of ovarian hormones over the menstrual cycle (Becker et al., 2005), women go through monthly hormonal shifts. The menstrual cycle is regulated via the hypothalamus-pituitary-gonadal axis with a prominent fluctuation of 17-β estradiol (E2) and progesterone (P4) levels. E2 and P4 are low in the early follicular phase. E2 reaches its peak right before ovulation (mid-cycle), while P4 remains low. P4-levels peak during the luteal phase, precisely in the mid-luteal phase, accompanied by a second, less prominent E2 rise. Both hormones reach low levels again by the onset of a new cycle. OCs, typically applied in a 21-day pill-on and a 7-day pill-off regimen, suppress the endogenous production of both, E2 and P4 resulting in steadily low levels of both hormones. Therefore, OC use has often been considered as a control condition for high-hormone phases during the natural menstrual cycle. However, OC use is not simply a state of low endogenous ovarian hormones: Synthetic ovarian hormones, i.e., ethinylestradiol and progestins, contained in the OCs, have the potential to modulate neuro-regulatory mechanisms involved in cognitive and emotional processing by interacting with E2- and P4- receptors in the brain (for review see: Montoya and Bos, 2017; Lewis et al., 2019; Brønnick et al., 2020). Thus, studies on the influence of OC use on emotional processing are informative in their own right.

Women also may go through cognitive and emotional shifts during the menstrual cycle: E2 and P4 contribute to cognitive and affective processes via acting on both, genomic (nuclear) and non-genomic (transmembrane) receptors in brain areas associated with emotion, memory, and executive functions (e.g., amygdala, hippocampus, and prefrontal cortex) (Gasbarri et al., 2012; Sundström-Poromaa, 2018). They also modulate synaptic transmission in the noradrenergic, dopaminergic, serotonergic, glutamatergic, and GABAergic systems and can thereby affect cognitive and affective processes (Toffoletto et al., 2014).

Areas of emotional processing that are often studied regarding the role of ovarian hormones are facial emotion recognition and emotional memory. *Facial emotion recognition* is indicated via accurately recognizing basic emotions from the facial expressions of protagonists, or complex emotions - requiring inferring mental states beyond the categorization of mere basic emotion (Baron-Cohen et al., 2001; Dziobek et al., 2008; Drimalla et al., 2019). This ability is essential in social cognition and indicated to be impaired in various psychiatric disorders (see: Sheaffer et al., 2009). Current research related to basic and complex emotion recognition suggests a sex effect where women tend to outperform men, although the results are quite mixed (Hoffmann et al., 2010; Wright et al., 2018; Wingenbach et al., 2018; Saylik et al., 2018; Connolly et al., 2019). Importantly, the link between facial emotion recognition and ovarian hormones is not straightforward. Limited evidence suggests that emotion recognition is enhanced during the follicular phase and impaired under OC use, whereas other studies fail to replicate these results or even reveal a better recognition of specific emotions under high P4 (vs. low P4) levels (for review see: Osório et al., 2018). *Emotional memory (or better emotionally biased memory)* is a relevant domain to examine the specific role of emotionally valenced (vs. neutral) material for memory consolidation and recall. Cognitive biases, including emotionally biased memory, are also a predictor of developing and maintaining affective disorders (Smith et al., 2018). Thus, understanding the role of ovarian hormones in emotionally biased memory has an important clinical relevance.

When reviewing menstrual cycle studies, it is important to consider what specific comparisons can tell us about the role of E2 and P4 in an emotional processing task. Assuming that cycle phases were identified correctly (see Becker et al., 2005; Sundström-Poromaa and Gingnell, 2014 for recommendations), different comparisons have different informational values. Studies comparing women during a wide range of days in the follicular phase with women during a wide range of days in the luteal phase can tell us something about the role of P4. Because, on average, the follicular phase would have low P4 and low to medium E2 – while the luteal phase would have, on average, high P4 and low to medium E2. However, it remains unclear if any reported effects are due to P4 or the specific combination of high P4 and medium E2 levels during the luteal phase. Also, comparing the luteal vs. the follicular phase using broad definitions of cycle days often will include some women tested during the pre-menstrual days and the actual days of menstruation. This adds potential menstrual or pre-menstrual discomfort as a confound. Studies trying to compare narrowly defined cycle phases deliver more information. For example, studies comparing the early follicular (low E2, low P4) with the late follicular phase (moderate to peak E2, with low P4) or with the ovulatory phase (peak E2, low P4) can provide evidence for the role of E2. Accompanied by a significant correlation between measured E2 levels and task performance, such a comparison delivers strong evidence for E2 modulation in a task. Comparing the mid-luteal phase (peak P4, moderate E2) to the late follicular phase (moderate to peak E2, with low P4) or with the ovulatory phase (peak E2, low P4) and the low-hormone early follicular phase can deliver strong evidence for a specific role of P4. Again, correlations with the measured hormone levels can strengthen this.

So far, reviews on the role of ovarian-hormone status address facial emotion recognition or emotional memory separately. In the present review, we update the state of the art of the corresponding research in both, facial emotion recognition *and* emotional memory. For both areas, we aim to find out whether hormone status is relevant in itself (main effects) or via interaction with stimulus features. Moreover, we intend to compare the result-pattern of both areas of emotion processing. Specifically, we hypothesize that high hormone states enhance emotional processing depending on the specific emotional valence of stimuli, i.e., we expect an interaction between hormone status and stimulus features. Additionally, we expect impaired emotional processing in OC users due to low endogenous ovarian hormones. Finally, we ask whether inconsistencies in the results may be associated with methodological differences among the studies.

## Materials and Methods

### Data Source and Search Strategy

We followed the guidelines of the *Preferred Reporting Items for Systematic Reviews and Meta-Analyses* (PRISMA) statement (Moher et al., 2009). The systematic literature search was performed using electronic databases *PubMed* and *Web of Science* covering all publications until September 4, 2020. We allocated studies in two areas: (1) emotion recognition and (2) emotional memory by using the MeSH terms (emotional OR emotion) AND (*X*) AND (estrogen OR progesterone OR menstrual cycle OR oral contraceptives) with (*X*) representing the separately searched areas of emotional processing, concretely (processing OR recognition OR empathy), and (memory OR recall) in the title and/or abstract. We noticed that some studies on facial-emotion-recognition can be found using the search term “empathy”. Therefore, we also added empathy to the search to avoid overlooking relevant papers.

### Publication Screening and Eligibility Criteria

After conducting the first selection of the search, we eliminated study duplicates. We organize the presentation of the search results based on the PICOS criteria. We specify the study design, population, interventions, comparators, and outcomes. Eligible studies had to examine healthy adult women in their reproductive age to restrict the influence of postmenopausal hormone conditions or physical and/or mental health conditions. Most studies compare the performance of free-cycling women in different phases of the menstrual cycle. Some studies also compare free-cycling women (overall) vs. OC users and free-cycling women vs. men. Only one study used a true experimental manipulation of the women’s hormone status by administering exogenous P4 (van Wingen et al., 2008). In the remaining studies, hormone status served as a quasi-experimental factor, relying on hormone levels and variations as naturally found across menstrual cycle phases and/or during OC use. In addition to comparisons between different hormone-status conditions, some of the emotional memory studies (10 of 21) included an experimental stressor vs. a non-stressful control condition as a between-subject factor. In these cases, experiments mainly used established stress-induction procedures such as the Cold Pressor Test (Hines and Brown, 1936) or the Trier Social Stress Test (Kirschbaum et al., 1993).

For studies on facial emotion recognition and emotional memory, the following criteria led to the inclusion of a study: (1) published in English, (2) examining healthy adult (18 or older) non-pregnant women in their reproductive age, and (3) including measurement or estimation of ovarian hormones (endogenous production or exogenous administration of E2 and/or P4) during the natural menstrual cycle and/or OC use. While available only in *PubMed*, the search was (4) limited to humans and (5) the search term being included in the title or abstract. No other limitations were applied.

## Results

### Search Result

**FIGURE 1:**
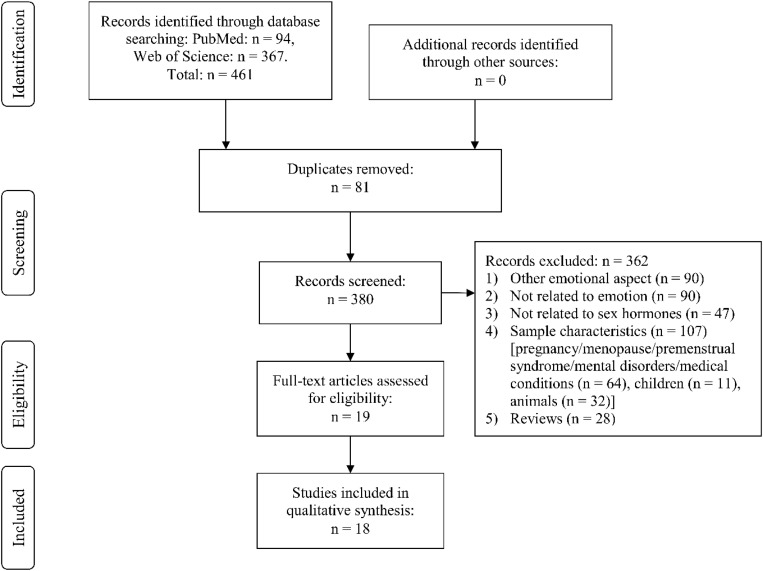
Flow chart showing detailed systematic search and selection process for emotion recognition.

**FIGURE 2:**
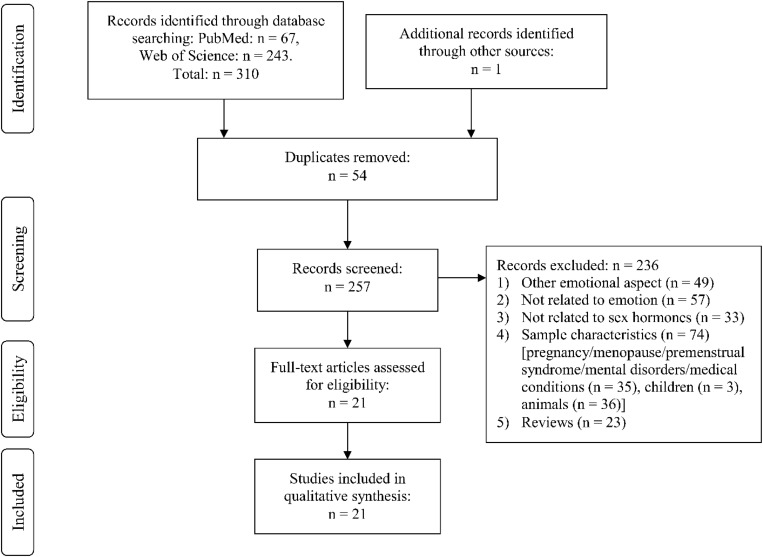
Flow chart showing detailed systematic search and selection process for emotional memory.

**FIGURE 3:**
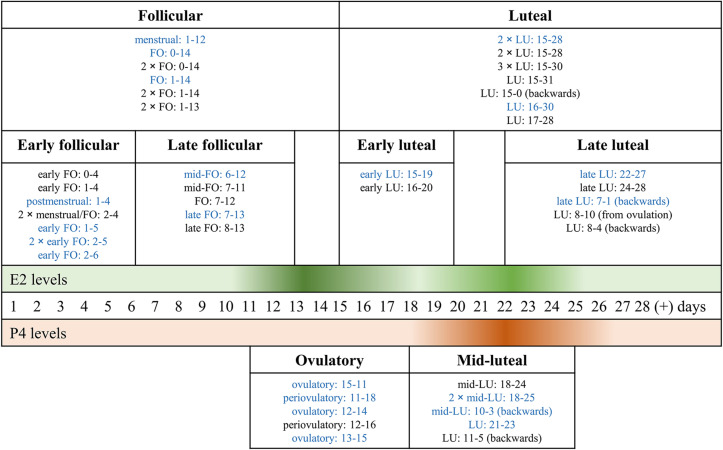
Classification of the definition of cycle phases across the reviewed menstrual cycle studies. The bold-face titles of the rectangular boxes show the labels we use throughout the review, to refer to phases of the menstrual cycle. The three horizontal stripes in the middle show the idealized 28 cycle days and the associated levels of 17β-estradiol (E2, green) and progesterone (P4, orange). The color gradient signifies hormone levels, with the highest levels shown in the darkest color and the lowest in the lightest color. The boxes are arranged so that the width of a box reflects the menstrual cycle days included under the specific label and the associated hormone levels. Entries in each box (small font) reflect the individual studies we combined under the same label, with their specific cycle days and their own cycle-phase labels. E.g., 2 × LU: 15-28 in the “Luteal”-box implies that two studies labeled cycle days from 15 to 28 days as the luteal phase. The figure serves to illustrate the variety of labels and included cycle days for the follicular and the luteal phase. Abbreviations. *E2*: 17β-estradiol; *FO*: follicular; *LU*: luteal; *P4*: progesterone. *[number] ×* …: number of studies using the referred labeling; *blue print:* emotion recognition studies; *black print:* emotional memory studies; *(+):* more than a 28-day cycle.

Through Web Search, we identified the following number of publications fulfilling the criteria described above. We first eliminated study duplicates. This resulted in 18 studies on emotion recognition (from initial 380 non-duplicate publications, with 362 excluded based on title and abstract, one of them after full-text assessment, Figure 1). For emotional memory, 21 publications (from initial 257 non-duplicate publications, 236 excluded) were included (Figure 2). The flow charts in Figures 1, 2 list the exact reasons for exclusion and the number of studies excluded based on a specific criterion. Figure 3 gives an overview of the definition of cycle phases across the reviewed studies.

### Study Characteristics

Tables 1, 2 contain the selected studies addressing facial emotion recognition, Tables 3, 4 summarize the corresponding data for emotional memory in healthy subjects. The tables account for the studies’ design, including the factor hormone status (predominantly realized as a between-subject factor, but in a few studies also as a within-subject factor, within a cycle phase, or as “pill on/off” phase in OC users) and at least one within-subject factor covering stimulus features (number of conditions in italics).

**TABLE 1:** Emotion recognition in hormone-status groups: Design and effects.

Author & year	Design	Group ME	Emotion type ME	Group × Emotion type	E2	P4	Compared groups
**Men vs. free-cycling women**							
Dan et al., 2019	3^1^ × *2*	no	no; [B]	−	−	−	Men ≈ lFO ≈ lLU
Guapo et al., 2009	4 × *7*	yes^*a*^	yes; [B]	yes	yes^–^	−	Men & mLU < eFO ≈ ovulatory
**Free-cycling women**							
Conway et al., 2007	2^1^ × *3*	yes^*b*^	yes; [B]	yes	−	−	low P4 < high P4
Derntl et al., 2013	2 × *6*	yes	yes; [B]	no	No	yes^–^	FO > LU
Derntl et al., 2008a	2 × *6*	yes	yes; [B]	no	no	yes^–^	FO > LU
Derntl et al., 2008b	2 × *6*	no	yes; [B]	no	no	no	FO ≈ LU
Kamboj et al., 2015	3 × *6*	no	yes; [B]	no	yes^–^	no	lFO ≈ eLU ≈ lLU
Pearson and Lewis, 2005	4 × *6*	yes^*c*^	yes; [B]	yes	−	−	eFO < lFO ≈ ovulatory ≈ LU
van Wingen et al., 2008	2^1^ × *2*	no	-; [B]	−	−	−	P4 ≈ placebo
Zhang et al., 2013	3^1^ × *6*	no	yes; [B]	no	−	−	lLU ≈ eFO ≈ ovulatory
**Oral contraceptive users vs. free-cycling women**							
Hamstra et al., 2014	2 × *6*	yes^*d*^	-; [B]	−	−	−	OC < FC
Hamstra et al., 2015	2 × *6*	yes^*e*^	-; [B]	−	−	−	OC < FC
Hamstra et al., 2016	3 × 6/2*	no	-; [B]/[C]	−	−	−	OC ≈ eFO ≈ mLU
Hamstra et al., 2017	2^1,2^ × 6/2*	yes^*f*^	-; [B]/[C]	−	−	−	OC < FC
Maner and Miller, 2014	4 × *4*	yes	yes; [B]	no	−	yes^+^	low P4 < high P4
Pahnke et al., 2019	2 × 2*	yes	yes; [C]	no	−	−	OC < FC
Radke and Derntl, 2016	2 × *6*	no	yes; [B]	no	−	−	OC ≈ FC
Shirazi et al., 2020	2^1^ × 3*	no	yes; [C]	no	no^*g*^	no^*g*^	OC ≈ FC

*eFO: early follicular phase; eLU: early luteal phase; Emotion type: basic emotions B (happiness, fear, sadness, anger, disgust and neutral), or complex emotions C (positive, negative); FC: free-cycling women; FO: follicular phase; lFO: late follicular phase; lLU: late luteal phase; LU: luteal phase; mLU: mid-luteal phase; OC: oral contraceptive users.*

*Notation of statistical analyses and effects: ME: main effect; yes: significant effect; no: no effect; -: not reported; × : interaction.*

*Effects for single emotions:*

*^a^: effect within anger;*

*^b^: when only anger and disgust included;*

*^c^: effect within fear;*

*^d^: within fear, anger, and disgust;*

*^e^: within anger and sadness;*

*^f^: within happiness and sadness;*

*^g^: measured in the FC group only, in the OC group testosterone was measured; E2: estradiol correlations; P4: progesterone correlations; yes^+^: significant positive association; yes ^–^: significant negative association.*

*^1^within-subject comparisons in the free-cycling women, i.e., comparing cycle phases.*

*^2^within-subject comparisons in oral contraceptive user, i.e., comparing pill-on and pill-off phase.*

*In the design-column, the italic values denote the number of levels of the within-subject factor.*

**TABLE 2:** Emotion recognition and ovarian hormones: Operationalization of the factors hormone status and emotion type.

Author & year	Hormone status	Stimuli for emotion type (images)	Task
**Men vs. free-cycling women**			
Dan et al., 2019	Men, lFO & lLU	happy, neutral, angry, fearful	Emotional face-matching task
Guapo et al., 2009	Men, eFO, ovulatory & mLU	anger, disgust, fear, happiness, sadness, surprise, neutral	FERT
**Free-cycling women**			
Conway et al., 2007	FC (low & high P4)	Fear, disgust, happiness	Self-constructed
Derntl et al., 2013	eFO & mLU	Anger, disgust, fear, happiness, sadness, neutral	VERT-K
Derntl et al., 2008a	FO & LU	Anger, disgust, fear, happiness, sadness, neutral	VERT-K
Derntl et al., 2008b	FO & LU	Anger, disgust, fear, happiness, sadness, neutral	VERT-K
Kamboj et al., 2015	lFO, eLU & lLU	Anger, disgust, fear, happiness, sadness, neutral	DEER-T
Pearson and Lewis, 2005	eFO, lFO, ovulatory & LU	Anger, disgust, fear, happiness, sadness, surprise	FERT
van Wingen et al., 2008	P4 vs. placebo in eFO	Anger, fear	Emotional face-matching task
Zhang et al., 2013	lLU, eFO & ovulatory	Anger, disgust, fear, happiness, sadness, neutral	CFAPS
**Oral contraceptive users vs. free-cycling women**			
Hamstra et al., 2014	OC & non-menstrual	Anger, disgust, fear, happiness, sadness, neutral	FERT
Hamstra et al., 2015	OC, FO & LU	Anger, disgust, fear, happiness, sadness, neutral	FERT
Hamstra et al., 2016	OC, eFO & mLU	Anger, disgust, fear, happiness, sadness, neutral; or Positive, negative	FERT; RMET
Hamstra et al., 2017	OC, eFO & mLU	Anger, disgust, fear, happiness, sadness, neutral; or Positive, negative	FERT; RMET
Maner and Miller, 2014	OC, lFO, ovulatory & LU	Anger, disgust, fear, sadness	FEI
Pahnke et al., 2019	OC, FO & LU	Positive, negative	RMET
Radke and Derntl, 2016	OC & FC	Anger, disgust, fear, happiness, sadness, neutral	VERT-K
Shirazi et al., 2020	OC, ovulatory & LU	Positive, negative	RMET

*CFAPS: Chinese Facial Affective Picture System; DEER-T: Dynamic Emotion Expression Recognition Task; eFO: early-follicular phase; eLU: early-luteal phase; FEI: Facial Expression Identification task; FERT: Pictures of Facial Affect Series; FO: follicular phase; KDEF: The Karolinska Directed Emotional Faces; lFO: late follicular phase; lLU: late-luteal phase; LU: luteal phase; mLU: mid-luteal phase; OC: oral contraceptive; RMET: Reading the Mind in the Eyes Test; VERT-K: Vienna Emotion Recognition Task – short version.*

**TABLE 3:** Emotional memory in hormone-status groups: Design and effects.

Author & year	Design	Group ME	Valence ME	Stressor ME	Group × valence × (stressor)	E2	P4	Compared groups
**Without experimental stress**								
Bayer et al., 2014	*2* × *3*	no	yes		yes (eFO × negative)	no	no	eFO ≈ mLU; negative: eFO > LU
Ertman et al., 2011	2 × *2*	yes	yes		no	no	yes^+^	Men ≈ FO ≈ LU*; emotional: FO < LU
Ferree and Cahill, 2009	3 × *2*	no/yes*	yes		−	−	−	FO < LU*
Ferree et al., 2011	2	no/yes*	−		−	no	no/yes^+^*	FO < LU*
Nielsen et al., 2011	2 × *2*	no	yes		no	−	−	OC ≈ FC
Nielsen et al., 2013b	3 × *2*	no	yes		no	−	−	Men ≈ FO ≈ LU
Petersen et al., 2015	2	yes**	−		−	−	−	OC < FC**
Pompili et al., 2016	2 × *3*	yes	yes		yes (ovulatory × positive)	yes^+^	no	eFO < ovulatory
Soni et al., 2013	3	yes*			−	no	no	lFO < eLU > lLU*
Spalek et al., 2019	2 × *3*	no	yes		yes (OC × positive/negative)	−	−	OC ≈ FC
Wassell et al., 2015	3 × *2*	no	yes		no	no	yes^+^	Men ≈ lFO ≈ mLU
**With experimental stress**								
Cheung et al., 2013	2 × *2* × 2	−	yes	−	yes (men × neutral × stressor)	yes^+^*	−	Men ≈ women
Felmingham et al., 2012	2 × *2* × 2	no	yes	no	yes (high P4 × negative × stressor)	−	−	mLU ≈ non-LU
Kuhlmann and Wolf, 2005	3 × *2* × 2	no	−	yes	−	−	−	OC ≈ FC
Maki et al., 2015	2 × *3* × *2*	−	yes	yes	yes (eFO × negative)	−	−	eFO ≈ lLU
Mordecai et al., 2017	2 × *3* × *2*	no	−	no	yes (negative & neutral x stressor)	−	−	OC (pill on ≈ off)
Nielsen et al., 2013a	2 × *2* × 3	no	yes	−	no	−	−	OC ≈ FC
Nielsen et al., 2014	2 × *2* × 2	no	yes	−	yes (FC × negative × stressor)	−	−	OC ≈ LU
Nielsen et al., 2015	3 × *3* × 3	−	−	−	yes (low E2 & P4 × negative)	yes^–^	no	OC, FO, LU
Schoofs and Wolf, 2009	3 × *2*	−	no	no	no	−	−	lLU
Zoladz et al., 2015	3 × *3* × 2	yes	yes	Yes	yes (FO/LU × high arousal × stressor/control)	−	−	FO < LU

*Group main effects describe short- or long-term recognition/free recall memory performance depending on the study if not noted otherwise; eFO: early follicular phase; FC: free-cycling women; FO: follicular phase; lFO: late follicular phase; lLU: late luteal phase; LU: luteal phase; mLU: mid-luteal phase; OC: oral contraceptive users.*

Notation of statistical analyses and effects: ME: main effect; no: no effect; -: not reported; × : interaction; yes: significant effect; yes^+^: significant positive association; yes^ –^: significant negative association. Factor valence covers pictures of either (1) negative valence only; (2) negative and neutral valences, or (3) negative, positive, and neutral valences. E2: estradiol correlations; P4: progesterone correlations; yes^+^: significant positive correlation; yes^ –^: significant negative correlation.

*intrusion frequency.

**recall of misinformation.

*In the design-column, the italic values denote the number of levels of the within-subject factor.*

**TABLE 4:** Emotional memory, ovarian hormones, and stress: Operationalization of the factors hormone status and stimuli (instrument and valence), and specification of the recall procedure.

Author & year	Hormone status	Stressor	Stimuli (Instrument, Valence)	Recall
**Without experimental stress**				
Bayer et al., 2014	eFO & mLU	−	IAPS (neutral, positive, negative)	48 hours
Ertman et al., 2011	FO & LU	−	IAPS (neutral, negative)	1 week
Ferree and Cahill, 2009	Men, FO & LU	−	Videos (neutral, negative)	48 hours
Ferree et al., 2011	FO & LU	−	Video (negative)	48 hours
Nielsen et al., 2011	OC & FC	−	Story (neutral, negative)	1 week
Nielsen et al., 2013b	Men, FO & LU	−	Story (neutral, negative)	1 week
Petersen et al., 2015	OC, lFO & LU	−	Negative images (misinformation [ ± ])	1 hour
Pompili et al., 2016	eFO & ovulatory	−	IAPS (neutral, positive, negative)	1 week
Soni et al., 2013	mFO, eLU & lLU	−	Film (negative)	72-hour diary
Spalek et al., 2019	OC vs. FC	−	IAPS (neutral, positive, negative)	10 minutes
Wassell et al., 2015	Men, lFO & mLU	−	IAPS (neutral, negative)	48 hours
**With experimental stress**				
Cheung et al., 2013	Men vs. Women	During encoding CPT (±);	IAPS (neutral, negative)	48 hours
Felmingham et al., 2012	mLU & non-LU	Post-encoding CPT (±);	IAPS (neutral, negative)	48 hours
Kuhlmann and Wolf, 2005	OC vs. eFO & LU	Post-encoding hydrocortisone vs. placebo	Words (neutral, negative)	5 hours
Maki et al., 2015	eFO & lLU	Pre-retrieval TSST (±);	Words (neutral, positive, negative)	24 hours
Mordecai et al., 2017	OC (pill on & off)	Pre-retrieval TSST (±);	Words (neutral, positive, negative)	24 hours
Nielsen et al., 2013a	OC & FO, LU	Post-encoding CPT (±);	IAPS (neutral, positive, negative)	1 week
Nielsen et al., 2014	OC & LU	Post-encoding CPT (±);	Story (neutral, emotional)	1 week
Nielsen et al., 2015	OC, FO & LU	Pre-encoding physical stressor (3 levels)	IAPS (neutral, positive, negative)	10-15 minutes
Schoofs and Wolf, 2009	lLU	Pre-retrieval TSST (±);	Words (neutral, positive, negative)	Immediate & 24 hours
Zoladz et al., 2015	Men, FO & LU	Post-encoding CPT (±);	Words (neutral, positive, negative)	Immediate & 24 hours

*CPT: Cold Pressor Test; eFO: early-follicular phase; eLU: early-luteal phase; FO: follicular phase; IAPS: International Affective Picture System (Lang et al., 2008); mFO: mid-follicular phase; mLU: mid-luteal phase; lFO: late-follicular phase; lLU: late-luteal phase; LU: luteal phase; OC: oral contraceptive users; TSST: Trier Social Stress Test.*

The factor hormone status was defined as phases during the menstrual cycle, or OC use, or men (low ovarian-hormone profile). Moreover, menstrual cycle studies use a range of different labels to refer to specific cycle phases. In some cases, the same range of cycle days receives a different label, depending on the study, while in other cases different cycle-day ranges are labeled identically (see: Figure 3, non-bold, small font entries). To aid understanding, we use the following labels (relative to an idealized 28-day cycle) throughout this review (given in bold font in Figure 3): Cycle days 1-14 after the onset of the menstrual cycle are labeled as the *follicular phase*. Within the follicular phase, days 1-6 are referred to as *early follicular*, and days 7-14 as the *late follicular phase*. The term *luteal phase* is used for days 15-28, with a subdivision into *early luteal phase* for cycle days 15-20, and *late luteal phase* for cycle days 22-28. As a special case, some studies use narrower phase definitions. These are labeled as an *ovulatory phase* (days 11-18, cycle length-dependent, verified by ovulation tests) and *mid-luteal phase* (days 18-24).

As for the within-subject factor, in emotion recognition studies (Tables 1, 2), it typically includes emotion type, mostly basic emotions (*n* = 16 of 18 studies). Only very few studies (n = 4 of 18) account for the recognition of complex emotions using the “Reading the Mind in the Eyes Test” (RMET; Baron-Cohen et al., 2001) with two of these four studies also measuring basic emotions. In emotional memory studies (Tables 3, 4), the within-subject factor covered emotional valence categories of negative (*n* = 3), additionally neutral (*n* = 10), or all three including positive valence (*n* = 8).

Emotional memory studies (Tables 3, 4) are additionally divided into those considering the quasi-experimental factor hormone status only (*n* = 11 of 21 studies), and those with additional experimental stress-induction (*n* = 10 of 21). The stressor mainly (*n* = 5 of 10 studies) consisted in the Cold Pressor Test (Hines and Brown, 1936) which is validated as an effective tool to induce a quick first-wave stress response with increased peripheral sympathetic activation and brain increase of monoaminergic transmitters. Further studies used a psychosocial stressor (Kirschbaum et al., 1993, *n* = 3 studies) with first- and second-wave stress responses (including a cortisol increase), a physical sympathetic stressor (handgrip, *n* = 1 study), and a pharmacological hydrocortisone administration (*n* = 1 study).

For all studies, we report the main effects of hormone status, stimulus features, and their interactions as well as correlations for E2 and P4 for emotion recognition (Table 1) and emotional memory studies (Table 3). We extend the analysis of main effects and interactions to the factor stress (in the case of memory studies). Table 2 (emotion recognition) and Table 4 (emotional memory) provide additional information about hormone-status groups, stimuli, and tasks used.

### Emotion Recognition and Hormone Status

The main effects of *hormone status* (Table 1) are reported in a few studies with a better emotion recognition in the follicular compared to the luteal phase (Derntl et al., 2008a, 2013; Guapo et al., 2009). An experimental P4-administration (400 mg vs. placebo) in the early-follicular phase increased the amygdala and decreased the fusiform gyrus activity (two brain sites critically involved in extracting emotional information from facial expressions) without affecting recognition accuracy (van Wingen et al., 2008). In contrast, high (vs. low) P4 levels were shown to be accompanied by better recognition accuracy (Maner and Miller, 2014) and stronger emotion intensity perception (Conway et al., 2007). Notably, these enhancing effects of P4 emerged when only negative emotions were considered (Conway et al., 2007; Maner and Miller, 2014). On the other hand, there were no changes in emotion recognition during the cycle phases with high E2 versus low/high P4 levels. E.g., there are null results when comparing between free-cycling women in their high-E2 late-follicular and/or ovulatory phase with women in their early follicular phase and/or different stages of their luteal phase (Derntl et al., 2008b; Zhang et al., 2013; Kamboj et al., 2015; Dan et al., 2019). OC use is consistently accompanied by impaired facial emotion recognition (Hamstra et al., 2014, 2015, 2016 [trendwise]; 2017; Pahnke et al., 2019). Although, these findings also have to face null results in small (Radke and Derntl, 2016) and large sample sizes (Shirazi et al., 2020).

Considering the *interaction between hormone status and single emotion type (or valence)* reveals whether the recognition of single emotions is facilitated vs. impaired in specific cycle phases. The majority of studies did not report (*n* = 6) or find (*n* = 9) any interactions. On the other hand, Guapo et al. (2009) found a better recognition of anger and sadness in free-cycling women in the early follicular phase as compared to the luteal phase and men. The ovulatory phase was associated with better recognition of fear as compared to men (Guapo et al., 2009). Similarly, in the ovulatory compared to the early follicular phase, women showed better recognition of fear (Pearson and Lewis, 2005). Besides, using a within-subject comparison, free-cycling women perceived more intense fear and tended to perceive stronger disgust in averted-gaze faces in their high-P4 stage, while there was no difference for happiness (Conway et al., 2007).

### Emotional Memory and Hormone Status

Emotional memory studies report fewer *hormone status* main effects (Table 3) compared to emotion recognition studies. Notably, emotion recognition studies often rely on testing one-way (or multivariate) ANOVAs for single emotions, whereas emotional memory studies usually use repeated-measures analyses including more than one valence category as a within-subject factor. Limited data show that memory recall was impaired in the follicular phase and enhanced in the ovulatory (Pompili et al., 2016) or luteal phases (Ertman et al., 2011; Zoladz et al., 2015). A reliable pattern emerges for intrusive content with more frequent intrusions in the luteal versus follicular phase (Ferree and Cahill, 2009; Ferree et al., 2011; Soni et al., 2013). Moreover, P4-levels, characteristic of the luteal phase, were predictive of mental imagery strength (Wassell et al., 2015). Regarding OC use, neither pill phase (on and off) (Mordecai et al., 2017), nor OC use per se (vs. free-cycling in the luteal or follicular phases) affected emotional memory (Kuhlmann and Wolf, 2005; Nielsen et al., 2011, 2013b, 2014). Nevertheless, OC use was accompanied by fewer false memories in a misinformation task as compared to free-cycling women (follicular and luteal) (Petersen et al., 2015). Moreover, *hormone status × valence interactions* were often not reported (*n* = 5) or found (*n* = 6). Available data show that women in the (early) follicular phase displayed a bias to recall negative content (Bayer et al., 2014; Maki et al., 2015), whereas in the ovulatory phase positive content was predominantly recalled (Pompili et al., 2016). In contrast, negative intrusions were more prominent in the luteal than follicular phase (Ferree and Cahill, 2009; Ferree et al., 2011). Interestingly, in a large sample (1215 OC users and 954 free-cycling women) OC users showed better recall of positive contents than free-cycling women (Spalek et al., 2019).

#### Stress Effects and Interactions in Emotional Memory

A *main effect of stress* (Table 3), where post-encoding stress improved delayed free recall, was only found in the minority of studies (Felmingham et al., 2012 [trendwise]; Kuhlmann and Wolf, 2005; Zoladz et al., 2015). In contrast, Maki et al. (2015) found better memory recall in the non-stressed group. A *stress × valence* interaction was reported (Mordecai et al., 2017) as an impairment of the recall of words of negative and neutral content in the stressed vs. control group (sample consisting exclusively of OC users). Likewise, Zoladz et al. (2015) showed that the stressed group recalled more negative words than the non-stressed controls. Stress affects women differentially in different cycle phases as *evident in three-way interactions* between *valence, hormone status, and stress*. Here data converge to show that stress experience after encoding leads to better negative memory recall during a high-E2 and high-P4-state in the free cycling women (vs. OC users), and similarly in the luteal (vs. follicular) phase (Felmingham et al., 2012; Nielsen et al., 2014, 2015). In contrast, using verbal material (words instead of images), Maki et al. (2015) found better memory for negative content in the follicular phase, and Zoladz et al. (2015) reported better recall of arousing (vs. non-arousing) words in the stressed (vs. not stressed) follicular group. On the other hand, Schoofs and Wolf (2009) and Nielsen et al. (2013a) did not find any stress-related interactions.

## Discussion

In the discussion, we address the role of hormone status in facial emotion recognition and emotional memory, discuss some of the methodological constraints of the reviewed studies, suggest some future directions, and finally name limitations of the present systematic review.

### The Role of Hormone Status

In both areas of emotional processing, i.e., emotion recognition and emotional memory, some common patterns can be identified. Not surprisingly, stimulus features, i.e., emotion type in case of emotion recognition, and emotional valence of the stimulus material significantly affect performance in both areas. The results are more complex and heterogeneous when accounting for the main effects of hormone status, and importantly its interaction with stimulus features. This is partly due to methodological differences between the studies regarding the selected cycle (sub-) phases. Moreover, even within studies on the same area of emotional processing (i.e., facial emotion recognition, and emotionally biased memory), the employed tasks and stimulus material varied.

Concerning *emotion recognition*, there is only limited evidence that the follicular phase with low E2- and P4-levels, on average, facilitates facial emotion recognition (Derntl et al., 2008a, 2013; Guapo et al., 2009). Accounting for the interaction with the valence of the recognized emotion (and thus a stimulus feature) reveals some facilitating role of P4 (higher in the luteal phase), particularly in recognition of negative emotions (Conway et al., 2007; Maner and Miller, 2014). However, the majority of studies addressing basic-emotion recognition do not find a difference between cycle phases of different P4-levels (Derntl et al., 2008b; Zhang et al., 2013; Kamboj et al., 2015; Dan et al., 2019). But some of these studies do not use the optimal cycle-phases to entail high P4-conditions, e.g., when the late luteal phase is used to account for a high-P4 stage (Zhang et al., 2013; Kamboj et al., 2015).

Reports of a facilitating role of P4 (higher in the luteal phase), particularly in recognition of *negative* emotions, are in line with enhanced *emotional memory* (Ertman et al., 2011; Zoladz et al., 2015) and negative intrusions (Ferree and Cahill, 2009; Ferree et al., 2011) in the luteal phase. There is also an interesting hint that it might be especially helpful to rely on the E2/P4 ratio when identifying stages of the higher vulnerability of intrusion frequencies (Soni et al., 2013): The early luteal phase (with initially low post-ovulatory E2 and relatively high P4) was shown to set a stage for more frequent intrusions in healthy women. On the brain-functional level, a bias of processing emotionally negatively-valenced information emerges under high P4-levels possibly due to increased amygdala reactivity in this hormonal state (van Wingen et al., 2008).

The luteal phase has recently been described as a “window of vulnerability” with increased brain network connectivity between the default-mode network and the salience network due to the actions of high levels of progesterone and its metabolite allopregnanolone but also because of the moderate estrogen levels (Andreano et al., 2018). This is accompanied by higher autonomic and stress reactivity, an increase in memory for negative events, a rise in negative affective symptoms (see: Andreano et al., 2018), and a higher risk of developing affective disorders during this cycle phase (Bryant et al., 2011). Future studies should find out whether the negativity bias might also favor a “better” recognition of negative emotions in a state of high P4 and moderate E2 levels. This would need a more sophisticated assessment of emotion recognition, not only relying on typically 4-5 basic negative emotions, and one positive emotion but by assessing an equal number of complex positive and negative emotions (see: “Methodological Constraints” and Limitations of the Present Review).

In correspondence with our expectations, OC use is rather associated with impaired emotion recognition (basic and complex) with no impact on emotional memory performance. In contrast, Radke and Derntl (2016) (in basic emotions) as well as a recent large-sample study (in complex emotions) (Shirazi et al., 2020) dismiss the impairing role of OCs in emotion recognition. Thus, OC effects on emotion recognition range from no influences to impairing effects. These contradicting results might come from a vast difference in the sample size and accordingly in the power of the studies. Moreover, inconsistencies may also be due to differences in the precision of subdividing the menstrual cycle phases of the free-cycling women that serve as a comparison group. OC intake is usually taken as a hormone state of low endogenous levels of E2 and P4. However, there is a difference between low levels of E2 and P4 in free-cycling women vs. in an OC condition (low endogenous levels of ovarian hormones). E.g., although synthetic ovarian hormones act on the same receptors as endogenous E2 and P4 (Jung-Hoffman and Kuhl, 1987), in general, there is evidence for many neurobiological differences between OC users and free-cycling women (regardless of cycle phase). OC use was shown to be accompanied with heightened emotional reactivity (depression, irritability), specifically in women prone to negative mood symptoms (Sundström-Poromaa and Segebladh, 2012; for review see: Montoya and Bos, 2017; Lewis et al., 2019). In adolescents, there is evidence for a higher prevalence of depression and suicide when taking OCs as compared to non-user adolescents, especially during the initial intake (debut) of the pill (for review see: Brønnick et al., 2020). OC use is further associated with structural and functional changes in areas involved in affective and cognitive processing such as the amygdala, hippocampus, prefrontal cortex, and cingulate gyrus (Brønnick et al., 2020). For example, there is data showing that gray-matter volume in prefrontal and hippocampal/parahippocampal regions is reduced in OC users compared to free-cycling women (Brønnick et al., 2020). In sum, uncovering the impact of OC use needs intensive research on its own.

### Methodological Constraints

*Facial emotion recognition:* The review consistently demonstrates the importance of stimulus features, namely valence of the stimulus material and/or emotion type. *Facial emotion recognition* (Table 2) varies with basic-emotion type (e.g., Derntl et al., 2008a, b, 2013; Guapo et al., 2009; Zhang et al., 2013; Kamboj et al., 2015) and/or valence of complex emotions (positive, negative, [neutral]) (e.g., Pahnke et al., 2019; Shirazi et al., 2020). In basic- as well as in complex-emotion recognition, positive emotions are identified with more accuracy than the negative ones (for basic emotions: Derntl et al., 2008a, b, 2013; Guapo et al., 2009; Zhang et al., 2013; Kamboj et al., 2015; for complex emotions: Pahnke et al., 2019). Current research on facial emotion recognition is predominantly restricted to basic emotions (*n* = 15), and only seldom on complex emotions (*n* = 4, Hamstra et al., 2016, 2017 [also assessing basic emotions]; Pahnke et al., 2019; Shirazi et al., 2020). Studies using basic emotions (happiness, fear, sadness, anger, disgust, [neutral]) to assess emotion recognition accuracy rely on one positive and typically 4 negative emotions. Using only one positive emotion (happiness) possibly leads to emotion type (valence) main effect and impairs understanding the role of hormone status in emotion recognition. Therefore, balancing out and representing positive and negative emotions is important to achieve comparability and avoid ceiling effects in performance. Since basic emotions do not offer multiple positive emotions, using a variety of complex emotions should be considered to provide this balance (e.g., via Multifaceted Empathy Test; Dziobek et al., 2008; Drimalla et al., 2019). Moreover, increasing the number of items for both types, positive and negative emotions, would improve the reliability of the measurement. Furthermore, concerning the recognition of basic emotions (16 of 18 studies here), we suggest reporting the validity and reliability of test instruments. E.g., in the 16 basic-emotion studies here, 6 different measurement instruments were used, and data on reliability and validity are not available (except for a 160-item version of the Vienna Emotion Recognition Task, VERT, Hoheisel and Kryspin-Exner, 2005). This will help to achieve a higher standardization and comparability of studies.

*Emotional memory:* While the bias of enhanced memory for emotional vs. neutral content is well known (e.g., Cahill et al., 1994), stimulus valence interacted with hormone status, supporting the relevance to be addressed in the present research field. In emotional memory tasks, only nine of the 21 studies include all three valences (negative, positive, neutral). Moreover, stimulus material used in these studies also differs between videos, words, and IAPS pictures. Here standardization should lead to assessing all three valence types (negative, positive, and neutral). Notably, the emotional memory tasks varied across the studies in several procedural features (see: Table 4), i.e., valence type, number, and order of stimuli, but also the temporal delay between encoding and recall of the material (10 min to 1 week), placement and variation of other factors such as stressor, etc. This makes it difficult to interpret the results. For example, if ovarian-hormone modulation of emotional memory is found, ideally one would want to disentangle the effect. Is it a long-term memory or a short-term memory effect? More studies including an immediate, a short-delay, and a long-delay retention test would address this problem. This would also inform us if ovarian hormones are involved preferentially in encoding or consolidation of emotional material (or both). Further, including material in different modalities (e.g., pictures, written words, spoken words) in every study could inform us if ovarian-hormone influence is global or modality and domain-specific. Also, the differences in hormone-status-group definition across the studies of both facial emotion recognition and emotionally biased memory might just indicate that varying outcomes come from the procedural diversity. Thus, it is reasonable to conduct more replication studies, instead of modifying study designs constantly to provide novelty.

### Quasi-Experimental Approach – Advantages and Further Suggestions

The quasi-experimental approach, as used in the vast majority of reviewed studies, holds the major advantage of *natural physiological* hormone fluctuations characteristic for the menstrual cycle (Gasbarri et al., 2012). However, it is worthwhile to choose cycle phases that represent a clear difference of E2 and P4 levels, i.e., by comparing the early follicular (low E2, P4) phase with the ovulatory (high E2) and with the mid-luteal (high P4, moderate E2) phase. OC users should be also included within the same study. Moreover, in the reviewed studies, hormone status was usually measured in a between-subject design by comparing subjects in different phases of the menstrual cycle (sometimes only self-reported and not validated with a hormone assessment). We strongly recommend considering the following points when comparing groups in different menstrual cycle phases: (1) proper tracking of the individual menstrual cycle in combination with hormonal assessment as recommended by Sundström-Poromaa and Gingnell (2014), (2) studying cycle phase effects preferably in a within-subject design, as it is fundamentally a within-subject process, and (3) defining narrower ranges within the follicular and luteal phase to account for the different combinations of E2 and P4 (Schmalenberger et al., 2021) (see Figure 3 for ranges used in reviewed studies). Even when all these three recommendations are met, comparing women in their natural cycle phases remains a quasi-experimental design that does not allow a causal interpretation of hormone-status effects since the subjects are not randomly assigned to the hormone-status groups. Thus, conducting experimental studies with administration of estradiol, progesterone, or a combination of both in the low-hormone windows during the menstrual cycle (i.e., in the early follicular phase) might be reasonable. E.g., exogenous E2 constituted a successful approach in fear-conditioning studies that revealed improved extinction recall (Graham and Milad, 2013).

### Future Research Questions

Addressing the role of sex hormones on emotion recognition and emotional memory also entails clinical implications. Sex differences in prevalence rates of affective disorders suggest that shifts in gonadal hormones are partly a contributing factor. E.g., women (compared to men) are twice as likely to develop affective disorders, such as depression and posttraumatic stress disorder, even when keeping the traumatic event constant (e.g., combat exposure in female and male soldiers, Luxton et al., 2010), or after accounting for trauma types by each sex (Ney et al., 2019). Interestingly, the risk of developing such disorders increases around puberty with the rise of sex hormones (Paus et al., 2008), or in the premenstrual, postpartum, and perimenopausal phases with low ovarian-hormone levels (Rapkin et al., 2002). Thus, understanding the role of ovarian-hormone status in emotional processing would result in important clinical implications by identifying stages of heightened vulnerability for the development of affective disorders (Andreano et al., 2018). Therefore, more research is needed in this direction.

Moreover, both, emotional learning and memory on the one hand (e.g. LaBar and Cabeza, 2006), and tasks requiring empathy and social recognition share several brain areas, i.e., hippocampus, amygdala, and prefrontal cortex (mainly ventromedial prefrontal cortex [vmPFC] and anterior cingulate cortex [ACC]). E.g., overall emotion recognition in the follicular phase (Derntl et al., 2008b) was related to amygdala activation but also ventral regions of the prefrontal cortex activated during fear and anger. The amygdala is a major site for *affective* empathy, especially in women (Derntl et al., 2010), but empathic abilities also include frontotemporal, occipital regions and brainstem areas (Derntl et al., 2010). Areas specific for cognitive empathy-related processes, also labeled as the theory of mind, cover the ventral temporoparietal junction (Kanske et al., 2015). Also, experimental administration of progesterone was effective in increasing amygdala reactivity, reducing activity in the fusiform gyrus, and impairing functional connectivity between the amygdala and fusiform cortex (van Wingen et al., 2008). Imaging studies addressing resting-state activity and functional connectivity are thus of further interest to contribute to the identification of brain areas specifically relevant for emotion recognition and empathy. Therefore, we further argue that research on emotion recognition should be extended. We suggest examining both, cognitive and affective empathy. While cognitive aspects of empathy cover the ability to infer the mental states of others (e.g., by recognizing and responding to facial expressions of emotion) and are at least partly included when assessing emotion recognition, assessing affective aspects of empathy is comparably rare in the area of ovarian-hormone research. Such aspects involve the observer’s emotional response to another individual’s emotional state (Dziobek et al., 2008; Drimalla et al., 2019). Therefore, investigating cognitive and affective empathy in different hormone-status groups as well as in OC use can provide more insights into the role of the ovarian hormones in emotional processing.

Furthermore, examining the role of other bodily signals, such as hand gestures for emotion recognition (e.g., Jospe et al., 2020), in addition to social information from facial expressions of the counterpart could be interesting to examine in women in different cycle phases as well.

Lastly, there are very recent systematic reviews on the brain sites responding to naturally fluctuating sex hormones during the female menstrual cycle (Dubol et al., 2021) and under contraceptive use (Brønnick et al., 2020). It is now time to relate the identified action sites to specific areas of emotional processing such as emotion recognition and emotional memory. We also suggest addressing an even broader scope of areas of emotional processing covering peripheral-physiological reactivity (such as skin-conductance responses) and mood assessment.

### Limitations of the Present Review

We decided to limit literature to healthy adult women in their reproductive age not exhibiting pregnancy or lactation. In contrast to other reviews on the role of ovarian hormones for selected emotion components, we here reviewed two emotion components, i.e., facial emotion recognition and emotional memory. In future reviews, it is worthwhile to consider additional age groups characterized by distinct concentrations of ovarian hormones, including puberty and menopause but also women during pregnancy and post-partum (see Osório et al., 2018 for facial emotion recognition). Moreover, other areas of emotional processing, i.e., emotion regulation, mood, fear and stress responses, and also reward sensitivity (Montoya and Bos, 2017) are of interest. Last but not least, clinical subgroups with anxiety and affective disorders could be also helpful to investigate. The underlying vulnerable affective states may demonstrate the role of ovarian hormones more intensely.

## Conclusion

There is evidence that variation in ovarian-hormone status – as evident in different phases of the natural menstrual cycle (i.e., E2 and P4 levels) – affects facial emotion recognition and emotional memory, especially while interacting with stimulus features. The reports quite consistently point to a negativity bias across emotion components with more intrusions and better memory for negative content during the luteal phase. This could be a potential indicator of higher vulnerability to developing affective disorders under these hormonal conditions. Notably, the quasi-experimental approach in ovarian-hormone research hinders any causal interpretation. Thus, more studies using an experimental administration of E2 and P4 should be considered. There is also questionable evidence for OC-related impairments in facial emotion recognition. Studies on facial emotion recognition and emotional memory addressing the role of ovarian-hormone status are quite diverse in methodology. Therefore, methodological consistency regarding tasks, hormone status validation, cycle phase definition, etc., is important. Further, we suggest extending the scope of facial emotion-recognition research to focus on complex instead of basic emotions.

By addressing the role of ovarian hormones for emotion processing, we also would like to extend the concept of “embodied emotions” to a hormonal perspective. The traditional concept of embodied emotions uses the manipulation of sensory (Williams and Bargh, 2008, as a traditional study), and motor input (Strack et al., 1988, for the initial study) and demonstrates that these manipulations affect subsequent interpersonal judgments of stimulus material or mood (for review see: Niedenthal, 2007; Wiswede et al., 2009; Fuchs and Koch, 2014). Similarly, we here suggest that bidirectional communication between peripheral steroid hormones and brain receptors in areas of emotional processing (including the amygdala, hippocampus, and prefrontal cortex) affects facial emotion recognition and emotional memory.

## Data Availability Statement

The original contributions presented in the study are included in the article, further inquiries can be directed to the corresponding author/s.

## Author Contributions

DG: investigation, formal analysis, writing – original draft, writing – review and editing, and visualization. MA: writing – review and editing and supervision. US: conceptualization, formal analysis, writing – review and editing, supervision, and funding acquisition. All authors contributed to the article and approved the submitted version.

## Conflict of Interest

The authors declare that the research was conducted in the absence of any commercial or financial relationships that could be construed as a potential conflict of interest.
